# Rehabilitation for Shoulder Instability – Current Approaches

**DOI:** 10.2174/1874325001711010957

**Published:** 2017-08-31

**Authors:** Anju Jaggi, Susan Alexander

**Affiliations:** RNOHT - Therapies Dept Brockley Hill Stanmore Middlesex Stanmore HA7 4LP United Kingdom

**Keywords:** Rehabilitation, Shoulder, Instability, Exercise, Assessment, Rotator Cuff

## Abstract

**Background:**

The shoulder relies predominantly on dynamic muscular control to provide stability. Successful treatment is highly dependent upon the correct clinical diagnosis, identification of anatomical structural defects and abnormal movement patterns so that rehabilitation programs can be designed accordingly and individualised to the patient.

**Method:**

A systematic outline is provided to guide the clinician on how to identify muscular insufficiencies both local to the shoulder joint and global muscles that can influence shoulder instability. Management is based on expert experience and current literature.

**Results:**

The Stanmore classification helps to correctly diagnose the type of instability and prioritise management. Symptom modification tests can help to guide management, however assessing individual muscle groups local to glenohumeral control is also recommended.

**Conclusion:**

Physical and psychosocial factors can influence motor control in the presence of pain and injury. A multi-disciplinary approach is required to avoid recurrence of symptoms with rehabilitation focusing on kinetic chain, scapular and gleno-humeral control.

## 1 INTRODUCTION

The aim of physiotherapy in the treatment of shoulder instability is to restore pain-free and normal motor control of the affected shoulder by using several distinct techniques that are applied in an appropriate and timely manner suited to the individual patient. Successful treatment is highly dependent upon the correct clinical diagnosis, identification of anatomical structural defects and abnormal movement patterns so that rehabilitation programs can be designed accordingly.

A thorough history is the fundamental starting point in understanding the type of instability that a patient may present with. Attention should be paid not just to the most recent episode of instability or indeed not be restricted to the shoulder joint itself, but should involve a more comprehensive approach of life-long incidence of apprehension, subluxations or voluntary dislocations of the shoulder, and evidence of generalised hyperlaxity in other joints. A failure to identify or address these factors may lead to failure of treatment or, in some cases, a deterioration of symptoms.

Several classification systems have been described to classify the different types of shoulder instability in order to guide treatment and it is dependent upon the skill and experience of the clinician to determine the correct diagnosis. Different parameters include the absence or intensity of trauma leading to the first episode of instability, the longevity of symptoms, presence of damage to anatomical structures and abnormal control of muscles that support and move the shoulder joint.

The Stanmore Triangle was described by Lewis *et al.* [1] who identified three main subgroups; Polar I-instability directly related to trauma with evidence of structural deficit within the glenohumeral joint, Polar II-atraumatic shoulder instability but also with evidence of structural deficit and Polar III – no structural defects and abnormal muscle control of the shoulder also referred to as muscle-patterning. The unique feature of the Stanmore approach is the recognition that the presentation of shoulder instability can change with time and prescribed treatment should be adapted accordingly to improve outcome. For example a patient may initially present with a shoulder dislocation following a high energy rugby tackle with an associated labral tear or Bankart lesion, however with time may develop recurrent instability that occurs with no provocation or even during sleep. This patient would initially be classified as Polar I but would then drift towards a Polar II group – this would be referred to as the I-II axis Fig. (**1**).

Surgical reconstruction is recommended in the presence of structural damage – Polar I group, and has been shown to prevent recurrence of shoulder instability [2]. The recommendation for Polar group III is to avoid surgery if there is evidence of muscle patterning and to adopt a more rehabilitative approach to correct the underlying muscle imbalance [3, 4]. Treatment of Polar II is controversial as often there is an element of muscle patterning that accompanies this presentation. Attention to detail in the history and examination is paramount in the II-III axis of the triangle to guide treatment, although the evidence for either surgery or therapy is lacking [5].

## 2 ASSESSING MOTOR CONTROL FUNCTION IN THE SHOULDER

The key elements in assessing shoulder instability should include posture and core stability, scapula control including function of the peri-scapular muscles and rotator cuff (RC) muscles, laxity of the joint, neurological and pain status, psychosocial factors including fear and anxiety to move the shoulder. Evaluation of each of these components will combine to form an overall impression that will determine whether surgical or non-operative management is preferred or indeed whether both should be considered and in which sequence [6]. Whilst some or several of these factors may contribute to the symptoms, it is important to decide which is the most dominant or the key ‘driver’ for the instability.

### 2.1 Muscles that Control Shoulder Movement

Muscles that control shoulder movement may be grouped into 3 distinct categories as listed below:

- **Muscles that Control Scapular Movement** Peri-scapular muscles include the upper fibres of trapezius, levator scapulae, which elevate the scapula, rhomboids major and minor which retract the scapula, lower fibres of trapezius which depresses the scapula, serratus anterior and pectoralis minor which protract the scapula.
- **Muscles that Act on the Humerus** Pecotralis major acts to depress the humeral head anteriorly whilst latissimus dorsi and teres major depresses the humeral head posteriorly with respect to the glenoid of the scapula.
- **Muscles directly related to Glenohumeral articulation** The four RC muscles act synergistically to compress the humeral head upon the glenoid cavity. The primary role of subscapularis is internal rotation, supraspinatus is elevation, and infraspinatus and teres minor externally rotate the arm. Boettcher *et al.* [7] used fine wire and surface electromyography (EMG) to record activity in several muscles around the shoulder girdle including the RC in healthy subjects. Each muscle was activated to varying degrees at different stages of each flexion-extension movement arc, dependant on the plane of movement. Increased activation of the posterior RC muscles was observed in forward flexion whereas increased activation of the anterior RC was observed when moving the arm into extension. In shoulder instability there is invariably dysfunction of some or all of these muscles and this may be due to structural disruption of the musculotendinous unit, poor proprioceptive sense, insufficient motor recruitment secondary to pain or disuse, excessive motor recruitment in an attempt to maintain stability, weakness of any component of the kinetic chain including the lower limbs, gluteal region, trunk and shoulder girdle.

## 3 PRINCIPLES OF ASSESSMENT

Assessment of the patient should include the basic principles of look, feel and move.

‘Look’ should start by asking the patient to stand upright with both extended arms resting by the side of the trunk. The normal relaxed posture should be noted. The ideal posture has been previously described in the literature and deviations from this could affect function at the shoulder [8]. Inspect for scoliosis or limb length discrepancy.The resting position of the scapula should be assessed to determine if it is equal or if there is prominence of the inferior pole or medial border suggesting protraction and rotation of the scapula [9, 10]. Testing for generalised laxity using the Beighton score [11] is useful to exclude underlying disorders of collagen that is associated with conditions such as Ehlers-Danlos syndrome, however more defined tests and the Brighton criteria [12] should be performed if there is clinical suspicion of collagen tissue disorders.

Lastly a screening test to evaluate the kinetic chain should be completed as the role of the lower limb and core stability correlate with upper limb function [13]. The ‘corkscrew’ test can be used by asking the patient to stand on one leg at a time and squat, to test overall stability. If the patient is seen to ‘corkscrew’ that is, twisting at the knee and hip the test is positive, exercises to improve lower limb and or core stability should be included in the first phase of rehabilitation [13, 14].

‘Feel’-the clavicle, acromioclavicular joint, acromion, coracoid, scapular spine, anterior and posterior glenohumeral joint lines should be palpated in sequence to elicit any tenderness.

Palpate for increased resting tone in the upper Trapezius fibres, Pectoralis Major and LatissimusDorsi, which may be indicative of abnormal motor recruitment patterns [15]. Palpation of the Rhomboids may be tender in patients with chronic protracted scapulae.

‘Move’ - Isolated scapula control may be observed by asking the patient to elevate and depress the scapula, by shrugging the shoulders up and down and then rotating the scapula forwards and backwards in clockwise and anti-clockwise directions. Quality and smoothness of movement and associated pain should be noted. If the patient is unable to perform these manoeuvres independently then they should be repeated whilst supporting the weight of the arm, if the movement improves (symptom modification) clinical experience has observed that the scapula may be a secondary compensatory strategy rather than the primary problem.

Further assessment of the scapula-thoracic muscles such as trapezius and serratus anterior function should be performed. Testing of the trapezius is best performed in prone positions with the patient holding the load of limb in varying degrees of elevation [16]. Serratus Anterior should be tested where the strength of isolated protraction can be performed such as supine lying or in sitting noting for increased winging when resistance is applied [16, 17]. (Figs. **2a** & **2b**).

Movement of the glenohumeral joint (GHJ) is examined by asking the patient to raise and lower their extended arms slowly in forward elevation and abduction at least three times to observe for muscular fatigue. This should be observed whilst standing behind the patient to record the range of movement and assess scapulothoracic movement. Any pain or apprehension should be noted.

External and internal rotation is performed with the elbow flexed to 90 degrees and the arm placed by the side of the trunk and then repeated with the arm abducted to 90 degrees. If any of the movements are abnormal or painful then symptom modification tests can be performed and forward elevation of the arms repeated. This can determine which dynamic motor components are contributing to the instability and can formulate the basis for prescription of exercises. Symptom modification tests include:1. Correcting general postural by standing up straight with the scapula retracted to neutral 2. Engaging the kinetic chain by standing on tiptoes, 3.Supporting the scapula during elevation, 4. Facilitating the rotator cuff and deltoid muscles by engaging the external rotators with resistance against the examiners hand through the movement [6, 10, 18]. From clinical experience if symptoms improve with tests 1 or 2 then therapy should include core stability exercises. If test 3 improves the symptoms then scapula control should be assessed. If test 4 improves the symptoms then the rotator cuff should be examined more thoroughly Fig. (**3**).

## 4 SPECIFIC INSTABILITY TESTS

Anterior/posterior Apprehension tests and labral tests can be useful to determine a diagnosis of instability and possible internal derangement [19] but lack the specificity as to the primary cause of the symptoms.

Gagey’s Hyperabduction test has been recognised as a useful test to evaluate the laxity of the inferior glenohumeral ligaments [20]. Anatomical and clinical findings indicate that the inferior glenohumeral ligaments control passive abduction at the glenohumeral joint. Passive abduction beyond 105 degrees is associated with lengthening and laxity of the inferior glenohumeral ligaments. If this is associated with a positive apprehension test then ligamentous laxity could be a driver to instability. The capsulolabral role in providing improved proprioception in the shoulder to aid motor control is still poorly understood and the role of surgery in improving this is not yet determined. It is still unclear whether surgical repair or reduction in capsular redundancy has any added value in improving patient’s symptoms compared to addressing the muscular stability alone particularly in those cases where there is minor damage or no bony injury [5] There are insufficient randomised clinical trials to examine the efficacy of exercise-based treatments in Polar II/III shoulder instability [21].

## 5 ASSESSING THE ROTATOR CUFF

If glenohumeral movement appears normal with no evidence of dyskinesia or if symptom modification tests fail to improve or aggravate symptoms then the authors would recommend a more detailed examination of the RC .

Recruitmentof the anterior and posterior healthy RC muscles should occur when the muscle is in its lengthened position (outer range) as well as its shortened position (inner range). Popular RC tests such as Belly-off test [22] and infraspinatus scapular retraction test [23] examine the rotator cuff muscles in their inner range, however it is equally important to assess RC activity throughout its full range, which includes outer range function. To test this the patient should lie supine with the elbow flexed to 90 degrees and the arm in 90 degrees of abduction. A small rolled towel should be placed under the arm to support its weight and prevent extension of the GHJ. Maximum active external rotation should be performed followed by resisted external rotation at the limit of the movement to test isometric strength with the posterior RC in its inner range and then resisted internal rotation in the same position to test the anterior RC in its outer range or lengthened position. The arm is then actively moved to maximum internal rotation,ensuring that the scapula does not lift off the from the couch, and resisted external rotation tests the posterior RC in outer range and resisted internal rotation in this position tests the anterior RC in its inner range. Use of a handheld Dynamometer to measure the RC strength in these positions has been shown to be a reliable method to gain more objective measurements [5, 24].

If there is poor recruitment or weakness of the RC muscles in any of these positions then the authors would recommend treatment is focused on improving the underactive component of the RC. It is important to recognise that shoulder girdle muscles work in synergy with varying patterns of activation and in order for one muscle to activate (agonist) the antagonist muscle must relax, therefore the inability of a muscle to recruit maybe secondary to inappropriate co-contraction of the antagonist muscle group. For example if there is reduced active external rotation this could be due to decreased co-contraction of the posterior RC and or decreased strength of the anterior RC in outer range. Treatment should initially focus on activation of the weaker muscle in positions where overactive muscles are more relaxed in order not to compensate Figs. (**4a** & **4b**).

## 6 REHABILITATING MOTOR CONTROL AT THE SHOULDER

Physiotherapy exercises for the shoulder use movement patterns that encompass the entire kinetic chain or those that specifically target selective motor control of the rotator cuff.

## 6.1 Early stage - Managing Apprehension and Pain

Pain, anxiety, fear and avoidance of movement are natural emotional reactions to shoulder instability,but are not necessarily correlated to severity of structural damage. In addition there is little evidence to advocate periods of immobilisation following both traumatic and atraumatic shoulder dislocations and early referral for rehabilitation to reassure and encourage normal motor control is recommended [25]. If the initial activation of the RC is compromised this may cause altered muscle recruitment patterns resulting in increased pain and instability. Early reassurance and focussed therapy is essential for patient recovery. Clinical skill is required to identify a safe zone in which the patient feels confident and happy to move their arm, or alterations in posture that make the shoulder feel more stable. Early activation of the RC and or recruitment of postural muscles could help in alleviating pain as well as to prevent compensatory strategies and over activation of aberrant muscle activity.

Shoulder exercises need to be considered into 2 categories; closed kinetic chain and open kinetic chain. Exercises which involve weight being placed through the arm with the hand fixed on a surface, closed chain exercises, facilitate co-contraction of the RC and deltoid muscles, thereby enhancing joint stability, stimulating muscular co-activation and proprioception [26, 27]. Open chain exercises are performed with no support to the hand and have increased loading at the shoulder due to the weight of the arm and the effect of gravity. Initially RC exercises should be performed with a stable body posture and the weight of the arm supported on a table, floor or wall to encourage muscular co-activation and scapula stability without increasing shear forces across the shoulder joint. Such positions may also provide guidance and reassurance to the patient as to where the shoulder feels more stable. In addition closed chain exercises have been shown to encourage recruitment of the scapula thoracic muscles, so if specific weakness has been found in middle and lower trapezius and or serratus anterior such exercises can be beneficial [28]. Early isometric RC exercises should also be encouraged Fig. (**5**) .

In those patients who are unable to gain selective RC recruitment due to poor trunk stability and poor scapula position, exercises to improve postural tone may be beneficial. Destabilising the base of support and challenging their postural muscles such as sitting on a Swiss ball standing on one leg or on a wobble board may encourage postural muscles to engage and reduce inappropriate over-activity of muscles such as Latissimus Dorsi Fig. (**6**).

A downwardly rotated scapula is commonly seen in patients with inferior or postero-inferior instability, encouraging scapula elevation in the movement pattern can be helpful in alleviating the instability [29]. In addition if RC and deltoid activation as a symptom modification test improves the stability, exercises to improve the recruitment of these muscles through the movement can be beneficial. Examples can be where the patient pushes outwards against a wall as they elevate the arm or pulling outwards against the resistance of theraband as they elevate the arm Fig. (**7**).

Previous studies have investigated the importance of the kinetic chain in shoulder function and it has been postulated that there is a correlation between lower limb and upper limb function [13]. Therefore if there is poor recruitment of the RC then a more stable base needs to be provided to optimise its recruitment pattern. This may be achieved by initially focussing on improving posture to stabilise the trunk and lower limbs.

## 6.2 Rehabilitation of the Scapular Muscles

Specific rehabilitation of scapula muscles would be required if selective weakness is identified and this appears to be the primary driver to the glenohumeral instability.Scapula imbalance is widely recognised in shoulder pathology and selective activation of the weaker muscle parts with minimal activity in the hyperactive muscles is key in resolving symptoms [28].

Observations found in overhead athletes with internal impingement and occult anterior instability have indicated dominance of upper trapezius activation in relation to middle, lower and serratus anterior function [29]. The work done by Cools *et al.* [28] has recommended 3 key exercises to gain optimal activation of middle and lower trapezius to minimal activation of upper trapezius. Based on their EMG analysis these exercises were side lying external rotation, side lying forward flexion, prone horizontal abduction with external rotation.

However in those patients who present with a drooping scapula and poor upward rotation increased activation of upper trapezius may be desirable. In the authors’ experience this is commonly observed in multi-directional instability [30]. If increased upper trapezius recruitment improves the instability then exercises with the arm above shoulder height in prone and side lying positions may be more preferable. In addition the modified scapula shrug exercise where the patient is encouraged to upwardly rotate the scapula in 30 degrees abduction compared to 0 degrees abduction encourages more upper trapezius activation and can be more effective in patients with multi-directional instability [31].

A commonly prescribed exercise to optimise the recruitment of serratus anterior is the ‘push up plus’exercise [32]. The exercise can be performed with the arm in weight bearing positions on a table or in four point kneeling, the patient is encouraged to push their chest away from the floor inorder to encourage scapula protraction activating serratus anterior in its inner range. The EMG recruitment of serratus anterior and trapezius can alter dependant on whether the exercise is performed on a stable or unstable base [33]. It has been suggested that performing the exercise on an unstable base encourages increased activation of trapezius where as a stable base encourages activation of serratus anterior. Therefore challenging the base of support i.e lifting the contra-lateral leg or opposite arm off the floor in four point kneeling may alter the scapula muscle recruitment patterns as well as performing the push up plus off an unstable base such as a swiss ball.

## 6.3 Rehabilitation of the Rotator Cuff

If weakness is identified in some or all of the RC muscles, rehabilitation should be directed at activating and strengthening the weaker muscles. Exercises can be performed in lying, sitting or standing postures dependent upon the ability of the patient.

If patients present with RC weakness in the outer range, or demonstrate scapula winging, lying supine is the preferred starting position to stabilise the trunk and scapula. With the elbow flexed to 90 degrees the patient is asked to rotate their arm though internal and external rotation, with the arm supported against gravity to reduce apprehension or anxiety and permit the arm to be moved through a wide arc of rotation with improved control. Once the patient can perform the exercise well, it should be repeated to the point of fatigue. In time, this exercise is gradually made more demanding by increasing the weight of the arm by either removing the underlying support, or by moving the arm into increasing degrees of abduction. These rotational exercises can be performed whilst lying prone again using towels to support the weight of the arm. This position improves inner range function of the RC but does require reasonable scapula control Fig. (**8a**).

Rotation exercises can be performed in the sitting position with the arm supported on a table, or the patient’s own leg (Fig. **8b**). Placing towels under the arm can provide extra support. If the elbow is elevated above the level of the shoulder, this decreases the weight of the limb and enhances compression of humeral head into the glenoid, therefore improving proprioception and confidence. It also encourages the patient to actively control the arm in positions that previously would have caused apprehension.

Once the patient can control the weight of their own arm through a reasonable arc of rotation external loads can be added. There is no evidence to guide treatment in terms of load, frequency or repetition [21]. It is recommended to empower the patient to determine these parameters in consultation with the therapist taking into account fatigue, pain and effort. The exercise needs to be achievable for the patient to perform, but not too easy so that it does not improve RC recruitment pattern. The focus should be on gaining stability in the deficient range with low loads rather than high loads in the competent range. In the authors’ experiencethe key to improve patient compliance is to prescribe a maximum of 2 specific exercises. It is better to perform 2 exercises twice a day properly rather than several exercises badly. Practice and repetition is more likely to enhance and maintain motor control function.

An example of a common exercise prescribed for anterior instability is placing the patient in prone lying with the arm fully supported and encouraging recruitment of the external rotators in inner range, as these muscles are often weaker in isometric testing. It also follows that these muscles primary role is to help prevent anterior displacement of the glenohumeral joint in elevation [7]. In the author’s experience most patients can achieve 8-10 repetitions performing up to 2-3 sets, twice a day and after 2 weeks of training can begin to feel an improvement.

Once the patient has achieved good activation and balance of the rotator cuff muscles, task specific functional exercises should be performed. Examples can include moving a ball clockwise/anti-clockwise on a wall, stabilising a ball on a wall, throwing and catching at speed, drop and catch, throwing at a target. Combining upper limb control with lower limb stability can start to incorporate patterns of muscle recruitment. Task specific activities have been shown to be effective in cognitive motor retraining [34]. Ultimately the patient needs to be challenged to regain confidence, if this is lacking the patient is at risk of developing compensatory strategies increasing the risk of re-injury.

## 6.4 Adjuncts to Motor Control Processing

If the patient is struggling to do basic exercises then 3 further modalities can be used; elastic therapeutic tape, biofeedback and functional electrical stimulation.

The use of tape remains controversial on its therapeutic effect and whether it alters proprioception but it is used widely in the clinical setting [35,36]. It can either be used to support the shoulder girdle providing the patient with a more secure feeling of the shoulder and therefore provide confidence and an ability to move or it can be used to help correct posture and position of the scapula providing feedback to the patient. When the patient moves out of the supported corrected position the tape can either become taut or slack depending on the method of taping guiding the patient on whether they are moving correctly or not. Surface electromyography to provide biofeedback can be another method to help guide the patient on which muscle groups they are using. For example a skin electrode overlying on the infraspinatus muscle and another on Latissimus Dorsi and through a visual display on a screen or audible feedback the patient can be encouraged to activate or inhibit one group of muscles over another. This form of real time biofeedback with repetitive practice has been shown to be effective in shoulder instability [37,38]. Functional electrical stimulation where muscles are directly stimulated by a muscle stimulator machine have been widely used to help restore more normal movement patterns particularly in the field of stroke and spinal cord rehabilitation however there is a paucity of evidence to their true efficacy and benefit in management of shoulder instability. It can be particularly effective in patients with more ingrained movement patters or with a degree of neurological injury. By placing direct muscle stimulation on underactive muscles it provides the feedforward sensory input the patient can then perceive and join in with. However, there is a lack of guidance or consensus on where and how such modalities should be applied. Clinically these techniques have been shown to have positive effects to help those patients who lack sensory awareness of their shoulder in space and in relation to the rest of their body or where abnormal habitual patterns of movement have been established.

## SUMMARY

Poor posture, weak core stability and dysfunctional motor control of the rotator cuff muscles or surrounding shoulder girdle muscles can compromise stability at the GHJ. It is important to adopt a multi-disciplinary team of clinicians to identify the underlying cause or main ‘driver’ of the shoulder instability. An appreciation of the impact of both physical and psychosocial factors that affect motor control strategies in the presence of pain and injury is required to avoid inappropriate management and risk of chronicity. A carefully managed progression of control of the kinetic chain, scapula position and GHJ movement offers the best solution for improving and sustaining outcomes in patients with shoulder instability.

## Figures and Tables

**Fig. (1):**
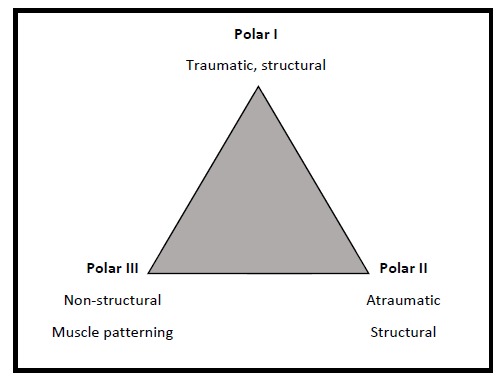
The Stanmore Instability Triangle.

**Fig. (2a):**
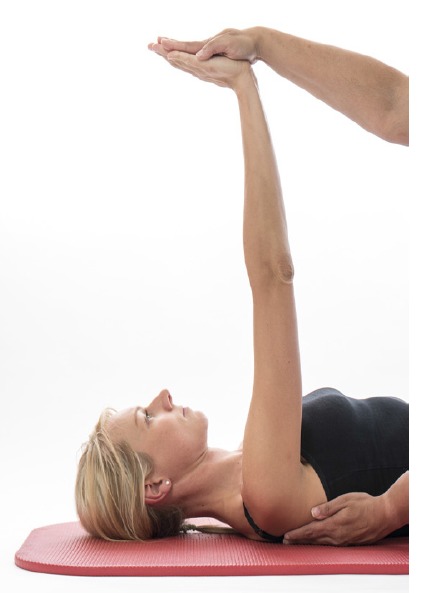
Serratus_Testing.

**Fig. (2b):**
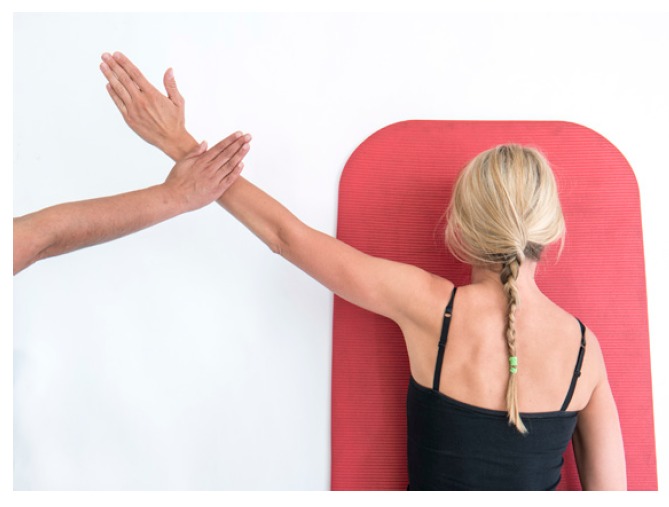
Trapezius_testing.

**Fig. (3):**
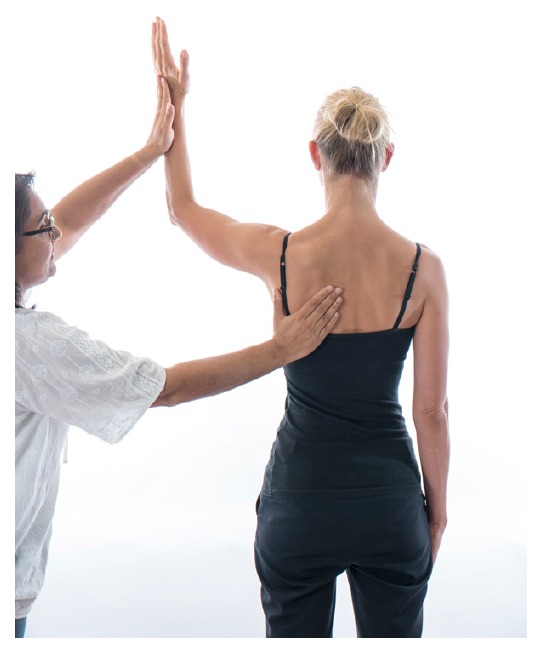
Scapula_and_cuff_facilitation.

**Fig. (4a):**
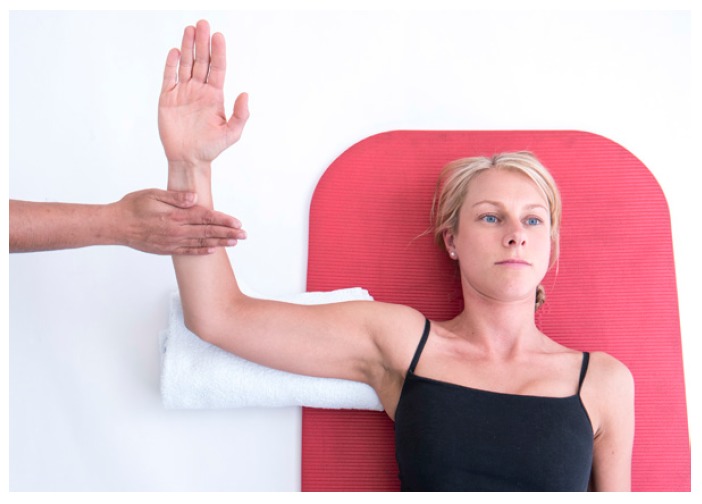
Resisted_Anterior_RC_testing.

**Fig. (4b):**
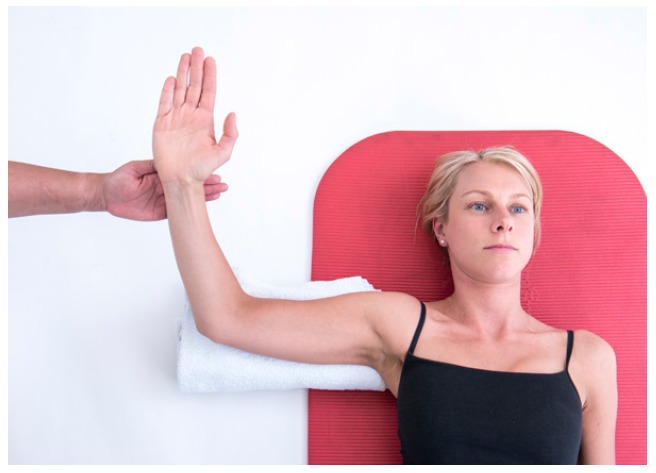
Resisted_Posterior_RC_Testing.

**Fig. (5):**
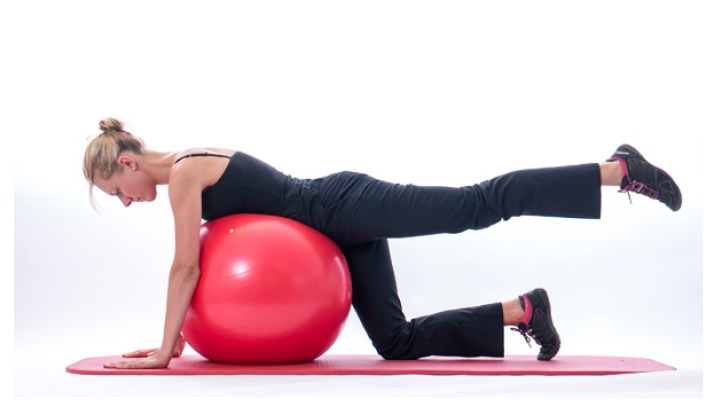
Closed_Chain_Exercise.

**Fig. (6):**
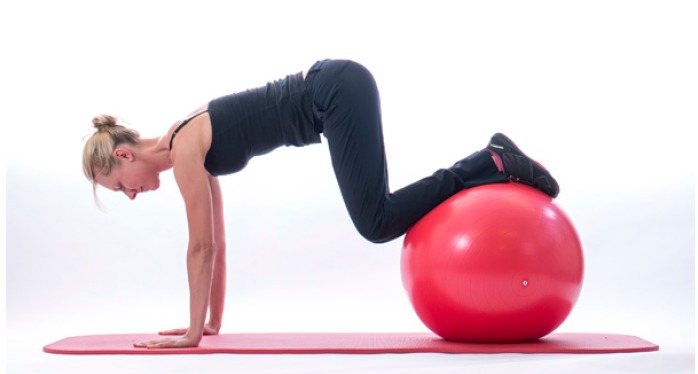
Core_stability.

**Fig. (7):**
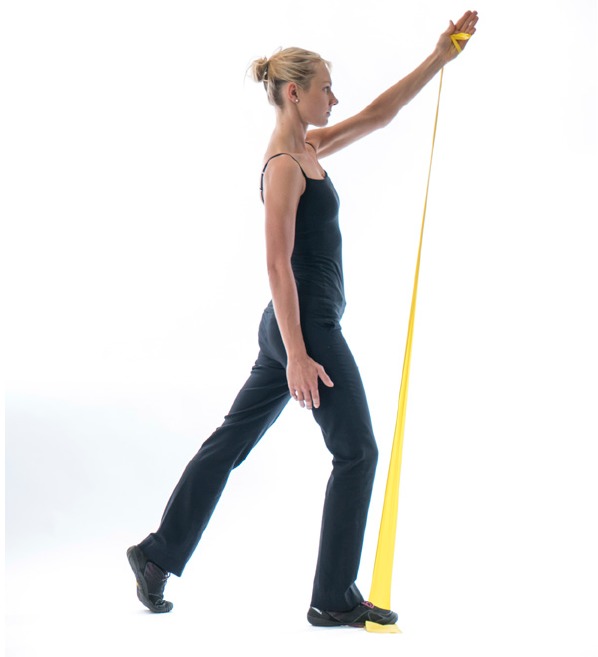
Resisted_RC_with_Kinetic_chain.

**Fig. (8a):**
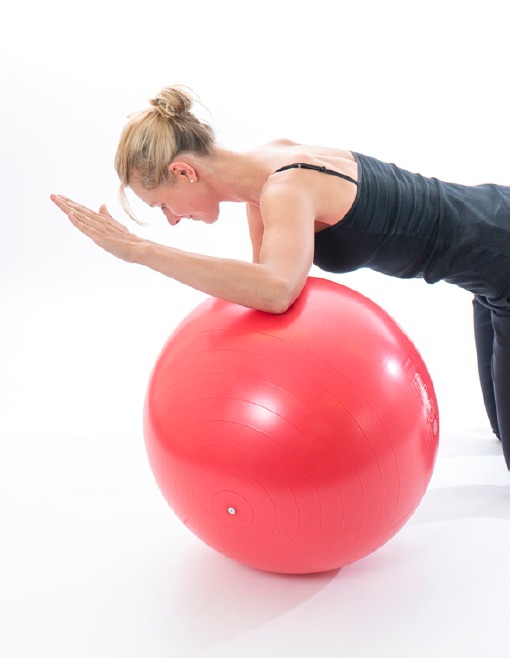
Post_RC_training_supported_prone_position.

**Fig. (8b):**
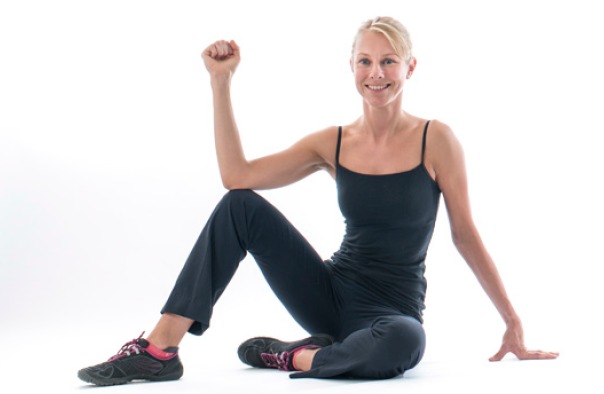
Supported_RC_training.
